# Factors influencing adult carer support planning for unpaid caregiving at the end of life in Scotland: Qualitative insights from triangulated interviews and focus groups

**DOI:** 10.1111/hsc.13472

**Published:** 2021-08-24

**Authors:** Susan Swan, Richard Meade, Debbie Cavers, Barbara Kimbell, Anna Lloyd, Emma Carduff

**Affiliations:** ^1^ Marie Curie Hospice Glasgow UK; ^2^ Marie Curie Edinburgh UK; ^3^ Usher Institute University of Edinburgh Old Medical School Edinburgh UK; ^4^ St Columba's Hospice Edinburgh UK

**Keywords:** caregiving, carer, palliative, planning, qualitative, support

## Abstract

Caring for a relative or friend at the end of life can be rewarding but all‐encompassing. These caregivers are often not identified, meaning their diverse needs remain unmet, and the lack of assessment, support and planning increases the likelihood of crisis and burnout. The Carers (Scotland) Act 2016 places responsibility on local authorities to implement such a plan, which will be fast‐tracked for carers supporting someone at the end of life. Our research described the factors which might influence this planning, triangulating primary focus groups with 15 carers and secondary qualitative data from transcripts with 30 carers, all of whom who had looked after someone with a terminal illness. Analysis was iterative, and constant comparative analysis of the secondary data informed the primary focus groups. Three main themes were identified; 1. The importance of early identification as a carer to enable timely assessment and support. 2. Carers experience isolation and loneliness which limits opportunities for support. 3. Responding in a timely fashion to carer assessment and support is vital to avoid crises. This research confirms that identifying carers early in the illness trajectory, ideally at diagnosis, is vital to avoid carer burnout. Health and social care providers have a key role in identification and should ensure, where possible, that carer needs are dynamically assessed, supported and documented. Finally, caring does not end after death, it extends into bereavement. Thus, we need to consider system and cultural change to ensure the experiences and needs of carers are addressed and valued.

**What is already known about the topic?**

- Identifying carers is challenging, because they do not necessarily see themselves as such or their needs as legitimate.
- There are few systematic ways for identifying carers for assessment and support.
- Support needs can be divided into those which support the carer to care, and the distinct needs of the carer themselves.

**What this study adds**

- Early identification of carers supporting someone with terminal illness is vital to avoid crises and burnout.
- Health and social care staff should proactively identify carers, acknowledge the role and inform them of their entitlements.
- Ongoing assessment at the key junctures of diagnosis, hospital admission, terminal decline and after death is advocated.

## 1 INTRODUCTION

In the United Kingdom, it is estimated that 500,000 of the approximately 6.5 million unpaid carers are caring for someone with a terminal illness (Grande et al., 2009). However, this is a difficult figure to accurately predict and could underestimate this ‘hidden’ subgroup of carers (Aoun et al., 2005; Burns et al., 2013). No specific figures are available for Scotland. These carers have diverse needs, spanning physical, psychological, social, spiritual, financial and informational domains. Home‐based caregiving can become overwhelming for the carer, resulting in a reluctance to leave the cared‐for person and a contraction of the carers' social interactions more generally. Moreover, there is often a progressive loss of sense of self and carers struggle with the competing demands of giving up work and juggling other family roles and relationships. All of this can reduce the likelihood of the carer seeking help (Carduff et al., 2016; Duggleby et al., 2017). Therefore, adequate and timely assessment and support is crucial (Carduff et al., 2014; Look et al., 2017; Reigada et al., 2015). The Scottish Government believed that both adult and young carers needed to be more consistently and better supported so they could continue to care (Scottish Government, 2015). As a result, Government passed legislation, The Carers (Scotland) Act 2016, which introduced a duty on local authorities to prepare an Adult Carer Support Plan (ACSP) or a Young Carer Statement (YCS) for anyone identified as a carer, or for any carer who requests one (Scottish Government, 2016). Recognising the importance and specific needs of those caring for someone at the end of life, during the progress of the legislation, the Government included a mechanism to allow the support plans for carers of people with a terminal illness to be fast‐tracked.

Palliative care is a holistic approach to caring for those approaching the end of life and their families (World Health Organisation, 2002). Identifying when a person is approaching their terminal phase can be challenging, especially for those with non‐malignant, unpredictable illness trajectories (Harrison et al., 2012) or frailty (Lloyd et al., 2016). Identifying carers is equally challenging, because they do not necessarily see themselves as such, nor do they see their support needs as legitimate (Carduff et al., 2014).

Traditionally, the focus of health care professionals has been on patients' needs, often missing those of the person caring for them. Largely, assessing carer need has remained ad hoc, unstructured and poorly documented by health professionals (Aoun et al., 2015; Aoun et al., 2017; Carduff et al., 2014; Ewing et al., 2016). A formal approach, such as the Scottish Government's ACSP or the Carer Support Needs Assessment Tool (CSNAT), provide opportunities for health and social care professionals (HSCPs) to build rapport with a carer, early in the illness trajectory, enabling better support later on (Røen et al., 2019).

The needs of those caring for someone with a terminal illness are diverse and include those which support the carer to care, and the distinct needs of the carer themselves, with the former being easier for the carer to define (Ewing & Grande, 2012). Research has shown that the timely identification, assessment and initiation of support can reduce the overwhelming pressure of caregiving (Aoun, Toye, et al., 2015), increase competence, sense of coping and satisfaction and decrease stress (Harrop et al., 2014). We were commissioned by the Scottish Government to describe factors influencing the creation of the ACSPs, for carers supporting someone with a terminal illness, under the Carers (Scotland) Act 2016. The aim of the project was to identify the needs that the ACSPs could meaningfully address for people providing unpaid care to someone in their final six months of life—which the legislation defined as a condition of receiving a fast‐tracked plan (Scottish Government, 2016).

## 2 METHODS

### 2.1 Design

The study triangulated data from two sources: (a) secondary analysis of interview and focus group transcripts with caregivers from studies conducted by the Primary Palliative Care Research Group, University of Edinburgh and (b) primary focus groups with bereaved carers, recruited through voluntary sector carers' centres in two Scottish cities. An iterative approach was used, with secondary analysis informing the topic guide for the primary focus groups. The researcher (SS) has experience of conducting qualitative research, and EC is an experienced qualitative palliative care researcher. The consolidated criteria for reporting qualitative research (COREQ) were used to report the study (Tong et al., 2007).

### 2.2 Ethics

Ethical approval for the study was granted by the University of Glasgow Research Ethics Committee (Number 200,170,027). All the studies used for the secondary analysis had appropriate institutional and NHS ethical approval. Written consent was obtained for all participants. All interview transcripts were fully anonymised.

### 2.3 Secondary analysis

#### 2.3.1 Context

The Primary Palliative Care Research Group, University of Edinburgh has a long history of conducting qualitative, longitudinal, multiperspective research with patients, informal caregivers and health professionals (Kendall et al., 2015; Murray et al., 2009). Longitudinal and multiperspective qualitative studies generate huge quantities of data and are considered an ethically robust way to generate new insights by ensuring research data are maximised (Irwin et al., 2012). Moreover, there is a growing imperative to use secondary analysis in rapid policy‐driven research, which is strengthened by including the voice of users (Ziebland & Hunt, 2014).

#### 2.3.2 Sample

Secondary data analysis was undertaken on anonymised transcripts from 19 bereavement interviews and 3 focus groups. The interview studies were conducted between 2006 and 2016 and used the same qualitative longitudinal, multiperspective methodology to explore the experiences and service usage of people living with different conditions in the last year of life and their carers: frailty (Lloyd et al., ,^2019^; Lloyd et al., ,^2016^), glioma (Cavers et al., 2012, 2013), advanced liver disease (Kimbell et al., 2015), colorectal cancer (Carduff et al., 2018) and stroke (Kendall et al., 2018). In each study, purposive sampling was used to reflect a range of age, sex and social deprivation in one health board in Scotland (see Table 1 for participant characteristics). All interviews were narrative in nature and used open‐ended questions to generate data on the experience of caring and bereavement. Having already published a secondary analysis of the patient data from this same repository, we knew that it was fit for the purpose of this research (Kendall et al., 2015).

**TABLE 1:** Secondary interview participant characteristics

Study code	Sex	Relationship to cared‐for person	Condition of cared‐for person
Frailty	F	Daughter	Frailty
Frailty	F	Daughter	Frailty
Frailty	M	Husband	Frailty
Glioma	F	Wife	Glioblastoma multiforme
Glioma	F	Wife	Glioblastoma multiforme
Glioma	M	Husband	Glioblastoma multiforme
Glioma	F	Wife	Glioblastoma multiforme
Glioma	M	Husband	Glioblastoma multiforme
Glioma	F	Wife	Glioblastoma multiforme
Glioma	M	Father	Glioblastoma multiforme
Glioma	F	Wife	Glioblastoma multiforme
Glioma	F	Wife	Glioblastoma multiforme
Colorectal ca	M	Husband	Metastatic colorectal cancer
Colorectal ca	F	Wife	Metastatic colorectal cancer
Colorectal ca	F	Sister	Metastatic colorectal cancer
Liver	F	Wife	Advanced liver disease
Liver	M	Husband	Advanced liver disease
Stroke	F	Daughter	Major stroke
Stroke	F	Daughter	Major stroke
Total number of carers		19	

In addition to the interview transcripts, the secondary analysis included three focus groups with a total of 15 participants, conducted as part of a funded project identifying carers of people at the end of life in general practice (Carduff et al., 2014, 2016). Participant characteristics for the focus groups are detailed in Table 2.

**TABLE 2:** Characteristics of participants from focus groups for secondary Focus Group (SFG)

Sex	Relationship to cared‐for person	Diagnosis of cared‐for person
F	Wife	Vascular disease
F	Wife	Cancer
F	Daughter	Alzheimer's disease
F	Wife	Stroke
M	Husband	Stroke
M	Husband	Multiple sclerosis
F	Wife	Multiple sclerosis
M	Husband	Dementia
F	Wife	Chronic obstructive airways disease (COPD)
F	Daughter	Dementia
F	Daughter	Dementia
F	Daughter	Dementia
F	Daughter	Cancer
F	Daughter	Frailty
F	Daughter	Alzheimer's disease
Total number of carers		15

#### 2.3.3 Analysis of secondary data

To ensure a clear understanding of the context of each study, the original studies' authors were consulted prior to commencing the secondary analysis and co‐authored this paper. Focus group and interview transcripts were thematically analysed (Braun & Clarke, 2006). Transcripts were read and re‐read to ensure familiarity with the data, and a long list of initial codes was derived from line by line coding of the text. These codes were then organised into broad categories, reviewed and where relevant, codes were grouped or merged into nodes. These nodes were defined and comparisons across, and relationships between categories were then explored. Knowing that the data were going to be triangulated with the primary focus group data, we did not apply global themes at this stage. Instead, we worked with the broad categories in a coding framework, which was used in the development of the topic guide for the primary focus group.

### 2.4 Primary focus groups

#### 2.4.1 Sample

Two focus groups were conducted with bereaved carers across Central Scotland in December 2017. Carers were recruited through voluntary sector carer organisations in two Scottish cities. Participants were purposively identified and invited to participate, by letter, through their carer organisation. The researcher telephoned those interested to answer questions and confirm the time of the focus group. All who indicated interest agreed to participate. Participant characteristics are detailed in Table 3.

**TABLE 3:** Primary Focus Group (PFG) participant characteristics

Focus group	Sex	Relationship to cared‐for person	Diagnosis of cared‐for person
PFG1	F	Husband	Dementia/cancer
	M	Son	Stroke/multi‐morbidity
	M	Husband	Cancer
	F	Daughter	Frailty
	F	Wife	Cancer
	F	Wife	Cancer
PFG 2	F	Wife	Stroke
	F	Daughter	Dementia/stroke
	F	Daughter/daughter in law/sister in law	Heart disease/dementia/cancer
	F	Daughter	Parkinson's disease/dementia
	M	Husband	Dementia/cancer
Total number of carers			11

#### 2.4.2 Data collection

Written consent was obtained by the researcher (SS) immediately prior to the focus group. Using an abductive approach, based on a review of the literature and the coding framework from the secondary analysis (Graneheim et al., 2017), a topic guide was developed. Participants were invited to share their experiences of caring, how, when and if they were identified as a carer, what support, if any, they received and of bereavement. The focus groups were conducted in the carers' centres and lasted 78 and 126 min, respectively. EC also attended the focus group to observe for areas of agreement or disagreement. The researchers (SS & EC) debriefed immediately following the focus groups. At the end of the project, participants received a lay summary of the findings.

#### 2.4.3 Analysis

Focus groups were audio‐recorded, transcribed verbatim and anonymised. A constructionist approach was used to identify areas of agreement and disagreement within the data (Barbour, 2007). Transcripts were analysed thematically, following the same line by line process and definitions of nodes as the secondary analysis (Braun & Clarke, 2006).

#### 2.4.4 Triangulating primary and secondary data

**FIGURE 1:**
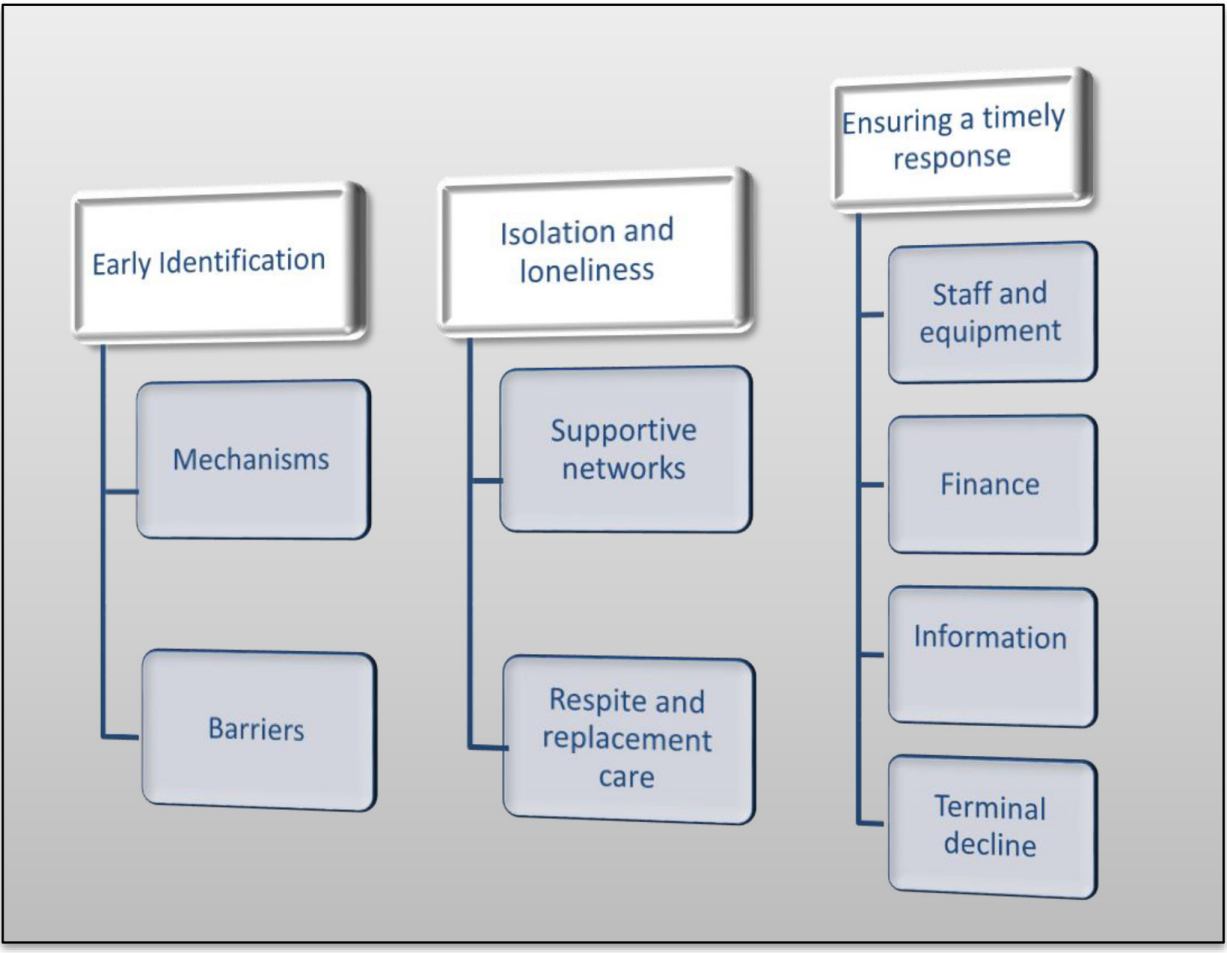
Conceptual map of the global themes and subthemes

The final nodes generated from analysis of the focus groups were compared and contrasted with the final nodes from the secondary analysis to triangulate the data and improve rigor and trustworthiness (Bryman, 2001). Where there were differences between the node frameworks, these nodes were discussed within the research team to agree a shared definition and understanding. For example, many carers spoke about the speed with which the cared‐for person declined and subsequently died. It became evident from the analysis that although death was expected, it usually happened unexpectedly. The researchers then returned to the secondary data to check this new category. This process of compare, contrast and discuss highlighted that ‘speed’ in general was an issue throughout a number of our nodes (e.g., information and communication, HSCP availability and response, financial support and the availability of equipment to support caring), which led us to the generation of global themes that we reviewed, defined and drafted into the final conceptual map of the global themes and subthemes, which is displayed in Figure 1. No data software was used to analyse the data.

### 2.5 Findings

The findings are derived from data from 45 carers. Three main themes were identified: (a) early identification, (b) isolation and loneliness and (c) ensuring a timely response. The timeliness of interventions, care and support was found to be an overlapping theme throughout, but the timing of planning and supportive interventions, as described in theme 3 was a key factor, with important implications for the Adult Carer Support Planning process and thus it is represented as a separate global theme.

### 2.6 Early identification

Mechanisms by which carers had been identified were raised in the primary and secondary focus groups only. Participants described interactions with different HSCPs, including General Practitioners (GP) and social workers, who were described as particularly important throughout the illness journey. Mechanisms to identification included both structured interventions, such as use of palliative care or illness registers in general practice, and opportunistic triggers, for example, automatic entitlement to the flu vaccination, which could lead to further opportunities for assessment and support.

Identification was challenging due to the gradual decline of the cared‐for person's independence. However, a change in health status, either of the cared‐for person or the carer and increasing dependence of the cared‐for person could lead to self and professional identification of the caregiver. Carer illness was a prompt to recognise the effect that caring was having:

*I started seeing myself as a carer when I was taken into hospital one night with a suspected heart attack because I was so stressed. [SFG, daughter, Alzheimer's disease]*

Barriers to identification were only discussed in the primary and secondary focus groups. They included the expectation and normalisation of the caring role as an extension of the familial relationship, where roles as family members or friends became extended or blurred and caring became ‘…part and parcel of life.’ [PFG, wife, cancer] and barriers such as HSCPs lack of understanding of eligibility for carer assessments and benefits.

Interventions to improve identification were suggested, with one carer encouraging professionals to consider that people requiring care at home might have lay people supporting them, who also require support.

*Maybe they should think about “does this patient need care and who's giving them that care? … And do THEY need care?” [SFG, daughter, Alzheimer's disease]*

### 2.7 Isolation and loneliness

#### 2.7.1 Supportive networks

Isolation was commonly described, influenced by the reluctance of the cared‐for person to involve others in their care and compounded by carers' overwhelming responsibility of having others depend on them. It was recognised that paid social care providers, attending to the physical needs of the cared‐for person, also gave emotional support to the carers themselves, who described depression, irritability and extreme fatigue as commonplace.

The focus group participants recognised the significance of carers' centres and peer support in reducing isolation through giving a ‘…sense of belonging’ [SFG, daughter, vascular dementia and visually impaired], legitimising their needs and empowering them. Being among peers was validating and allowed carers to openly express their emotions. One carer spoke of this as both difficult and beneficial:

*… But I was still emotionally, I don't know how to put it. I was still fragile, is that how you say it?… But I must admit, up there, with everybody up there [at the carers' centre], they were great with me, and they helped me a lot [PFG, husband, dementia/laryngeal cancer]*

Participants described that familial relationships were sometimes complex and strained, particularly as the cared‐for person approached the end of life. Some carers preferred support from an impartial person such as a HSCP during and after the caring experience. Others felt well supported by friends and family:

*My sister was at the end of a phone, so when I had a “kick the filing cabinet” moment after I'd had a particularly challenging day, I could phone her up and sometimes there would be complete silence from her perspective, as I tried to get my breath back [PFG, daughter, Parkinson's disease/dementia]*

Those caring for someone with a diagnosis involving confusion or personality changes, such as a brain tumour or dementia, alluded to anticipatory grief and spoke of managing distressing behavioural issues:

*I was very, very upset, very difficult, because it wasn't [caregivers' husband] [Glioblastoma multiforme, wife]*

The data highlighted the significant role of primary care professionals in supporting carers, particularly when the cared‐for person neared the end of life. Varying levels of support from GPs and District Nurses (DN) were described, largely regarding the provision of symptom management, but also in terms of general emotional support and having someone to answer carers' questions. Generally, experiences were positive, yet three carers reported feeling let down by a lack of GP input, with one saying ‘…he never saw a doctor [GP] at all’ [Glioblastoma multiforme, wife]. Likewise, empathetic care from hospital and care home staff was praised and when lacking, missed.

Having an assertive HSCP to coordinate in times of crisis was welcomed. Crises could occur suddenly or result from a build‐up of issues and could trigger acknowledgment that additional help was required:

*It's the bashing your head against a brick wall, it's going from crisis, to crisis, to crisis. And knowing, not knowing when the next crisis is coming, not knowing what that crisis is going to be, but knowing that it's coming. [PFG, daughter, Parkinson's disease/dementia]*

Across the data set, consistency and a high‐quality service were recognised as important, with frustration expressed when failures occurred.

Feelings of isolation were particularly prevalent when the person had died. Many carers described sadness when few HSCPs offered condolences, leading to a general sense of abandonment. Some carers spoke of being comforted by social carers and ongoing GP support into bereavement. Carers who had attended carers' centres described the benefits of continuing to do so following bereavement through maintaining peer support:

*The carers need care now, you know, the people that's, you know, been through it*. *They're still raw and tender. [PFG, husband, lung cancer]*

#### 2.7.2 Respite and replacement care

The sense of responsibility to provide round‐the‐clock care caused carers anxiety. They also worried about becoming unwell themselves. Time away from the caring role was widely discussed as being significant in allowing the carer to ‘be a better person’ [SFG, daughter, Alzheimer's disease] and maintain their physical and emotional health:

*I was thinking back over the week, and thinking, what did I do right, and what didn't I do right and what do I do now? And I always found that that space, three hours, with good coffee, and a catching up with myself, was really another lifesaver. [PFG, husband, dementia/cancer]*

Sleep disturbance was common and described as lowering the threshold for coping. Carers described sleeping in the same room as the cared‐for person but being frequently woken and fearful of going to sleep. Basic human needs were affected:

*I couldn't even go to the toilet, when I went to go to the toilet she was screaming. So her and I sat up all night, me in one chair and her in the other. [Frailty, daughter]*

Overall, input from professional carers, through replacement care, gave a sense of security but was not without challenges. When away from the cared‐for person, carers described feeling anxious and watching the clock. Additionally, cared‐for individuals were reluctant to be left with someone else, preventing the carer from accessing much needed support.

### 2.8 Ensuring a timely response

The intensity of support that carers required varied, but the importance of delivering support at the right time was unanimous. There was agreement that in the final days to weeks of life, frequent input from GPs and DNs was beneficial, with one carer reporting that the GP was visiting them at home every day:

*Then (GP) came in every day. I mean that was over four days, but that felt like weeks. [Liver, wife]*

DN visits were perceived as reassuring, helpful for triaging concerns and liaising with the GP, however was detrimental when the caregivers' needs were not recognised:

*…they [District Nurses] didn't recognise what a terrible life I was having and I was so busy and I was so heart‐broken with them. [SFG, wife, stroke]*

Many carers, particularly those caring for a person with a malignant condition, described the terminal decline as faster than anticipated. Many talked about this lasting a few months or days, coinciding with an increase in their own supportive needs. Unexpected events, such as a stroke and falls, resulted in an immediate change in condition and subsequent carer needs. Proactive advance care planning was described as helpful to avoid inappropriate interventions, such as resuscitation.

Carers described having received a professional estimate of prognosis, but as it was often inaccurate, was distressing:

*And then my own doctor, [name of doctor], she came in a couple of days after to see how I was coping, and she says, “we didn't expect it so quick” so whatever happened in the end I don't know. [Colorectal cancer, wife]*

A quick decline was described as negatively affecting the carer's ability to process and adapt to the evolving situation.

Likewise, the speed of availability of HSCPs and support equipment, such as commodes, was found to be significant. Conversely, late or absent input magnified the burden on the carer, sometimes having to purchase equipment themselves, because they did not know how to access it or there was a lack of availability when needed:

*The commode I had, had nae [no] wheels on it. Er, [patient] went into [hospice] and what did they bring me? A commode with wheels on it. I thought, it's too late. [Glioblastoma multiforme, wife]*

Similarly, carers were unsure of what financial support they were entitled to, denying them of financial benefits, with one saying he ‘didn't even know what carer's allowance was.’ [SFG, husband, multiple sclerosis]. Accessing financial support often required professional assistance, and it was important that benefits and grants were received quickly.

Communication between professionals was crucial in terms of accessing specialist advice and avoiding repeatedly having to answer the same questions:

*And I remember that first day, there was a procession of people I didn't know coming to see me, and they were all asking the same questions, and I was absolutely shattered [exhausted]. [PFG, wife, stroke]*

Timing of information‐giving was significant. Many carers reported that they would have welcomed earlier information and advice. Information regarding accessing support, services and prognosis was obtained from a wide range of sources, through signposting from HSCPs and self‐directed through phone lines, information booklets and the internet.

The all‐encompassing nature of caring in addition to not knowing who to call for help or advice left carers feeling unsupported:

*…maybe it was my state of mind at the time, I don't know, I wasn't thinking straight—I didn't even think “Where can I go for help?” [SFG, wife, peripheral artery disease]*

Concern was also raised by one carer about how quickly help would arrive once a call was made.

## 3 DISCUSSION

We set out to describe support needs of carers who are caring for someone in the last 6 months, which might influence the uptake and completion of the fast‐tracked ACSP in Scotland. The findings highlighted the importance of formally identifying carers. Simply, support cannot be offered if the carer is not recognised. Thus, identifying carers early, and enabling crisis prevention, is the vital first step. Identifying carers early should reduce pressure on the fast‐track system for the ACSP in Scotland, as a plan will be in place before the cared‐for person enters the last six months of life. Projections of palliative care provision show that, if current trends continue, 66% of Scots will die at home, in a care home, or in a hospice by 2040, which has implications for unpaid carers who will be required to provide much of the support (Finucane et al., 2019). Similarly, demand for palliative care and support in England and Wales is expected to rise by between 25% and 47% in response to increasing multimorbidity, cancer and dementia deaths and the aging population (Etkind et al., 2017).

Our findings highlight that HSCPs are ideally placed to identify carers. We now know there are many opportunities for identifying patients for a palliative approach, for example frequent hospital admissions or visits to the GP, but that this does not always happen (Harrison et al., 2012; Mason et al., 2015). For example, frail older people and their carers often miss out on supportive interventions, because there is no diagnosis of frailty (Lloyd et al., ,^2019^; Lloyd et al., ,^2016^). Similarly, Poole et al. (2018) found that patients and carers with dementia did not recognise the condition as being palliative, which affected the likelihood of discussions about planning and future needs taking place. Therefore, it is vital that future efforts to ensure equitable access to carer support include carers supporting people with all conditions (Poole et al., 2018).

In their caring role, carers come in to contact with many HSCPs, but missed opportunities to identify carers remain. Our findings highlight that HSCPs, particularly those in primary care settings, are considered vital in identifying and supporting carers. We advocate that HSCPs make a presumption that people have an informal carer and that they are offered documented identification (i.e., on an Electronic Palliative Care Co‐ordination System) (Finucane et al., 2020) and needs assessment. Importantly, this approach would also alert health professionals to those patients who do not have a caregiver at home, which may be an important factor in future planning, particularly regarding preference for place of death. Our findings also illustrate that identifying carers need not be complicated—identifying a patient as being someone in need of support should prompt consideration of the role and needs of their unpaid supportive network. However, the complex, relational and collaborative nature of caring can lead to tensions between patients, unpaid caregivers, family members and formal caregivers. Poole et al. (2018) found that patients with dementia, their unpaid caregivers and health care professionals, placed differing value on future planning. Research highlights that the relationship between the family and their HSCP is essential, as poor communication can be synonymous with poor co‐ordination (Morris et al., 2015; Ventura et al., 2014).

Hospital discharge is another opportunity and can be a difficult transition for carers. Carer assessment should be conducted during admission, not discharge, from hospital, to allow adequate time to discuss and plan how best to meet need, sustain caring at home and minimise readmissions (Ewing et al., 2018; Totman et al., 2015). To do this, staff need to be aware of resources for signposting, have discharge meetings with the patient, carer and relevant specialist and community staff and feel confident to engage in conversations (Ewing et al., 2018; Røen et al., 2019).

The carers in this study reported varying support needs across the illness journey, extending to death and bereavement, illustrating their evolving need over a long trajectory. Thus, ongoing, dynamic assessment, with suggested prompts for reassessment being diagnosis, admission to hospital, terminal decline and after the cared‐for person's death, is advocated. It is vital that pre‐bereavement needs are met, particularly emotional support to build carer resilience and reduce isolation (Totman et al., 2015) and promote good health post death (Nielsen et al., 2017; Roper et al., 2019). When family support is lacking, carer resilience is lower, but provision of emotional and informational support from health care professionals can improve resilience (Roper et al., 2019).

The rapidity and unpredictability of terminal decline, with health care professionals often struggling to determine a definite prognosis, highlights the necessity of early access to carer assessment, information provision, honest conversation and signposting. If there is rapid decline in a person whose carer has not been identified, or for carers of people with a late diagnosis, rapid provision of support for the carer will be vital. Our findings suggest that help may not be required or desired until late in the disease process, but knowing who, how and what to access is crucial.

Isolation was found to span all the domains of need: physical, psychological and social. Health care professionals need to be aware of the potential for loneliness, as carers struggle to maintain existing relationships, hobbies and sometimes paid employment. Burden of care and isolation can increase as the cared‐for person declines, through to bereavement (Hudson et al., 2018). Addressing this is complex and, as this study demonstrated, complicated by the impact of who individuals wanted as their caregiver and to what extent. This could increase the burden on the carer, who had limited opportunities to address the isolation they were experiencing. Additionally, carers may be living with their own health conditions, thus increasing the complexity of the caring situation. Caring can change the relationship between carer and cared‐for person, which in turn can increase loneliness. This is particularly true for illnesses affecting personality and mental capacity. HSCPs can minimise this, in part, through providing support and advice that reduces the sense of responsibility that accompanies caring, enabling timely access to equipment and communicating difficult information around prognosis sensitively (Totman et al., 2015). This study highlighted the important role played by primary care professionals; others have reported GPs' desire to be actively involved in supporting carers at home (Røen et al., 2019). However, caution must be taken to ensure seamless care across the primary and secondary settings.

The impact of loneliness and isolation of carers should be addressed through a wider public health approach. The publication of the Scottish Government's social isolation and loneliness strategy demonstrates a commitment to this important issue (Scottish Government, 2018). By targeting this consequence of caring, carers' physical and mental health can be improved, creating a more positive caring experience. This will require innovative thinking and likely an improved focus on use of volunteers to support carers in the home environment (Abel, 2018; Caswell et al., 2019). The compassionate communities/cities movement emerging across the globe recognises that health and wellbeing promotion, when done well, must include carers as well as patients (http://www.compassionatecommunities.org.uk/). A civic response must include help with practical support such as shopping, cleaning and transport as well as an emotional and social response. Health care professionals have a role in recognising and mobilising the wider supportive networks of carers to ensure they feel, and are supported, to care (Abel, 2018).

## 4 STRENGTHS AND LIMITATIONS

This study was focused in one country but involved a large data set that triangulated qualitative primary focus groups and secondary interview data. The caregivers cared for individuals with diverse malignant and non‐malignant conditions, providing a range of experiences and support. One challenge of conducting secondary data analysis is that the original studies' aims and objectives are unlikely to perfectly match those of the primary study. In this project, there were more relevant illustrative quotes from the primary sources, glioblastoma multiforme interviews and frailty bereavement interviews; however, all data sources informed the overall narrative of the study. A potential limitation is that the secondary data are from 2006 onwards, and thus, caution should be applied when interpreting it. However, as the data are related to lived experience, many treatment and pathways are the same, and as the secondary data were able to be synthesised with the primary, the data are comparable and relevant. Another potential limitation is that the caregivers in the primary focus groups were recruited from third sector carer organisations and had positive experiences of carers' centres. Both primary and secondary data were collected across urban populations in Central Scotland, meaning the experiences of caregivers in rural locations, or those at a distance from hospice or tertiary centres, were not captured. The primary and secondary data were generated from retrospective accounts of experience and are subject to recall bias and may not necessarily capture changing needs across the trajectory. However, it was a strength that we captured meaningful data on the importance of support continuing into the bereavement period.

## 5 CONCLUSION

Identifying unpaid carers supporting someone approaching the end of life is complex. Lack of support for the caregiver leaves both carer and cared‐for at risk of a breakdown in the care provided, and in need of a rapid response if the caring role is to be sustained. This study has highlighted the importance of identifying carers early, to ensure they have the support they need. We advocate for the need to move towards the presumption that a patient has an unpaid carer supporting them at home and ensure they are aware of their entitlements and how to access them. Assessment of support needs should start early and finish late in the carer's journey, with the process having a longer trajectory than currently recognised, into bereavement. The fluctuating nature of terminal decline results in a dynamic experience for carers, which necessitates frequent reassessment to determine if support needs have changed. It is encouraging that the Scottish Government has prioritised and legislated for carer planning. This is the first step in making real change, where system and culture do not focus solely on the patient, at the expense of the carer, enabling services to continue to provide high quality patient and carer centred end‐of‐life care.

## CONFLICT OF INTEREST

The authors have no conflict of interest to declare.

## AUTHOR CONTRIBUTIONS

SS, EC and RM designed the study. EC acted as project lead. Primary data collection was undertaken by SS and EC. Data used for secondary analysis was collected by EC, DC, BK and AL. Primary and secondary data analysis was conducted by SS and EC. Themes were discussed and finalised by the wider team. The manuscript was initially prepared by SS and EC, with input from RM, DC, BK and AL. All authors reviewed and gave final approval of the version to be published.

## Data Availability

The data that support the findings of this study are available from the corresponding author upon reasonable request.
